# Spectroscopic Behaviour of Copper(II) Complexes Containing 2-Hydroxyphenones

**DOI:** 10.3390/molecules27186033

**Published:** 2022-09-16

**Authors:** Emmie Chiyindiko, Ernst H. G. Langner, Jeanet Conradie

**Affiliations:** 1 Department of Chemistry, University of the Free State, P.O. Box 339, Bloemfontein 9300, South Africa; 2 Department of Chemistry, UiT—The Arctic University of Norway, N-9037 Tromsø, Norway

**Keywords:** 2-hydroxybenzophenone, copper(II), TDDFT, dye-sensitized solar cells (DSSCs)

## Abstract

Theoretical investigations by density functional theory (DFT) and time-dependent DFT (TD-DFT) methods shed light on how the type of ligand or attached groups influence the electronic structure, absorption spectrum, electron excitation, and intramolecular and interfacial electron transfer of the Cu(II) complexes under study. The findings provide new insight into the designing and screening of high-performance dyes for dye-sensitized solar cells (DSSCs).

## 1. Introduction

Copper(II/I) redox shuttles and copper dyes have received much interest as redox couples and dye sensitizers, respectively, for dye-sensitized solar cells (DSSCs) applications in recent years [1]. These first examples of full-copper solar cells, using copper(I) dyes with a copper(II/I) redox shuttle [2,3], create a new path for cheap and environmentally friendly DSSCs. Especially 4-coordinated copper(I) complexes, containing polypyridyl ligands, showed promising properties as dyes in DSSC [4,5,6,7].

**Scheme 1:**
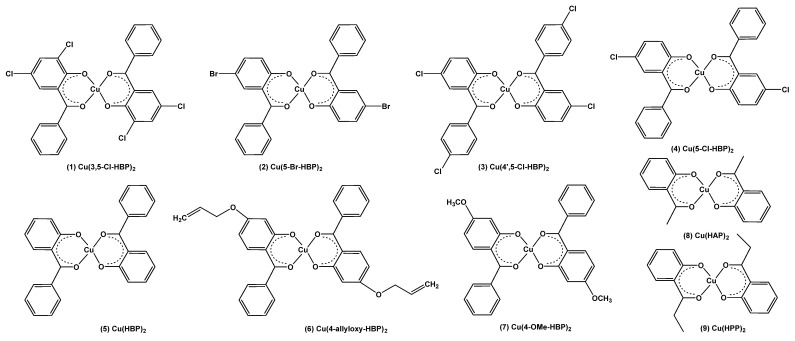
Structure and numbering of the Cu(L)_2_ complexes of this study. L = 2-hydroxyphenone ligand.

Theoretical calculations using density functional theory are a handy tool to predict the UV-vis properties of both organic (for example LEG4 [8] and phenothiazine based dyes [9]) and metal-containing dyes. DFT calculations are reported for different transition metal complexes suitable to be used as dyes in DSSC, for example the well-known experimentally efficient Zn-porphyrin based sensitizer YD2-o-C8 [10], the well-known N3 ruthenium(II) sensitizer [5], and copper(II) complexes containing bipyridine- and phenanthroline-based ligands [1]. During such theoretical studies, UV/vis spectra are computed [11,12], absorbance maxima in the solar range are identified, and the molecular orbitals (MOs) involved in excitations relating to the absorbance maxima identified. Knowledge of the MOs involved in excitations can identify the type of excitation such as metal-to-ligand charge-transfer (MLCT) [11,13,14], ligand-to-metal charge-transfer (LMCT), ILCT (intra-ligand charge transfer) [12], and ligand-to-ligand charge transfer (LLCT) bands [9]. DFT calculated properties to evaluate the dye performance in DSSCs, such as charge transfer characteristics, light harvesting efficiency (LHE), the excited-state lifetime (τ), driving force of electron injection (ΔG_inject_) and dye regeneration (ΔG_regenerate_), are often reported [5,11,13,14,15,16,17]. DFT calculations can assist in the design of complexes with improved photo conversion. We recently reported on the experimental synthesis and electrochemical properties of a series of 4-coordinated copper(II) complexes containing 2-hydroxyphenones. Since 4-coordinated copper(I) complexes, containing polypyridyl ligands, showed promising properties as dye, in this contribution we focus on the UV-vis spectroscopic properties of 4-coordinated copper(II) complexes containing 2-hydroxyphenones (see Scheme 1). Results obtained by density functional theory (DFT) and time-dependent DFT (TD-DFT) methods are theoretically evaluated to provide a new insight into the designing and screening of high-performance dyes for DSSCs. 

## 2. Results and Discussion

**Figure 1:**
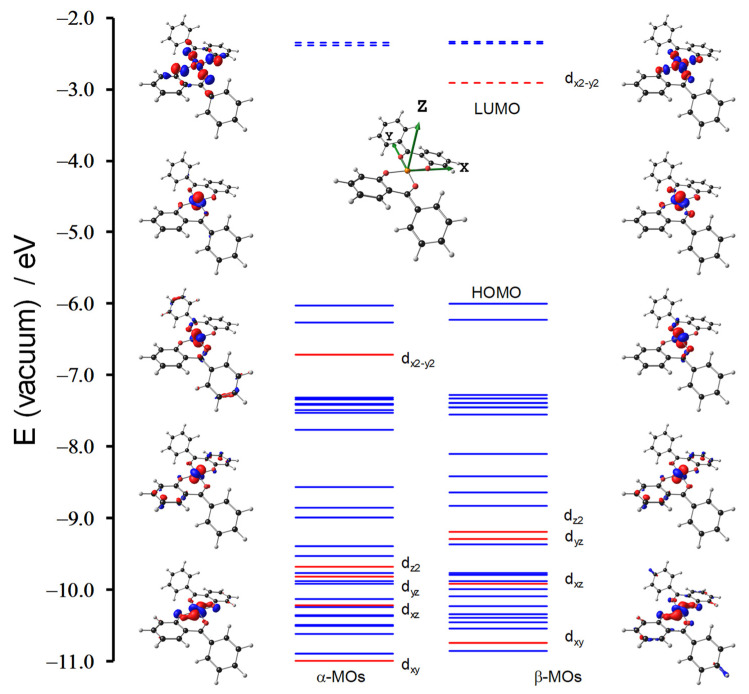
B3LYP/6-311G(d,p)/def2-TZVPP calculated Kohn−Sham MO energy level diagram of Cu(HBP)_2_ (**5**) with the mainly copper-d-based MOs shown. The energy of occupied and unoccupied MO energy levels is shown with solid and dotted lines, respectively. Mainly copper-d-based and ligand-based MOs are shown in red and blue, respectively. A contour of 0.06 e/Å^3^ was used for the orbital plot. Colour code of atoms (online version): Cu (orange), C (grey), O (red), H (white).

The Cu(II) complexes of this study are d^9^ complexes of d-electron occupation $d_{xy}^{2}d_{xz}^{2}d_{yz}^{2}d_{z^{2}}^{2}d_{x^{2}-y^{2}}^{1}$; see Figure 1 for a molecular orbital (MO) energy level diagram of Cu(HBP)_2_ (**5**) with the mainly d-based and ligand-based MO energy levels identified, showing the mainly d-based MOs. Since the HOMO is ligand-based and the LUMO copper-based, the maximum wavelength UV-vis excitation peak of Cu(HBP)_2_, (**5**), will be of ligand-to-metal charge- transfer (LMCT) character. Low energy UV-vis excitation peaks of Cu(HBP)_2_, (**5**), that do not involve the LUMO, will be ligand-to-ligand charge transfer (LLCT) character.

### 2.1. Experimental and Calculated UV-Vis

**Figure 2:**
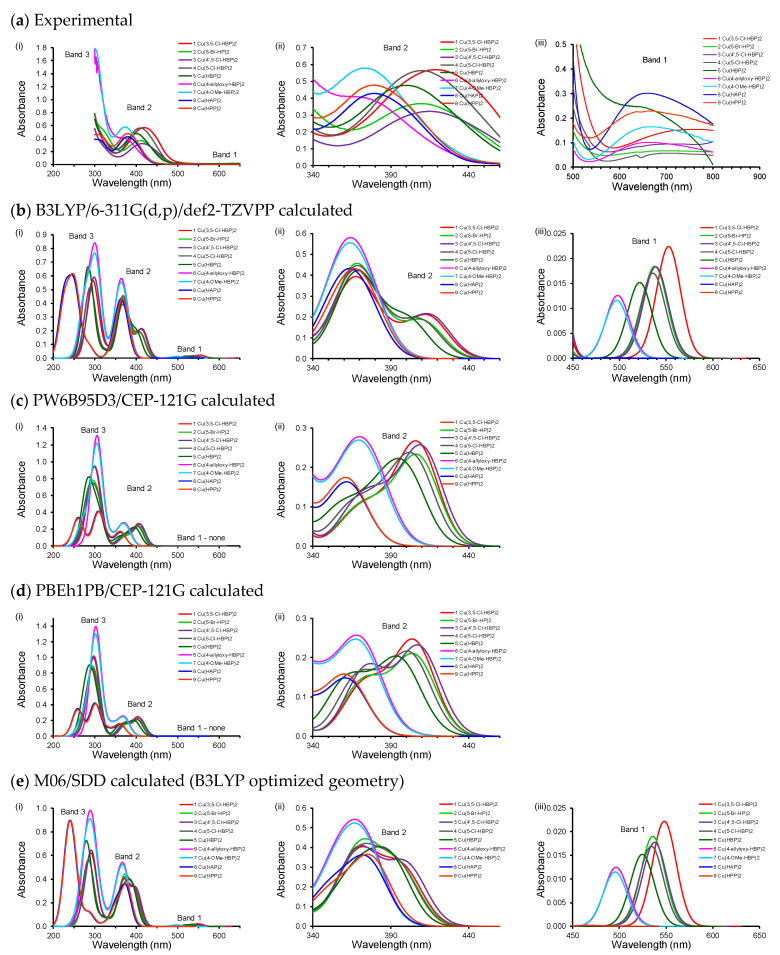
(**a**) Experimental absorption spectra of the Cu(II) complexes (**1**)–(**9**) in DMSO in region (**i**) 300–650 nm, (**ii**) 340–450 nm and (**iii**) 500–800 nm; (**b**–**e**): Simulated absorption spectra of the Cu(II) complexes (**1**)–(**9**), calculated with the (**b**) B3LYP/6-311G(d,p)/def2-TZVPP, (**c**) PW6B95D3/CEP-121G, (**d**) PBEh1PB/CEP-121G and (**d**) M06/SDD methods, all using DMSO as implicit solvent.

**Figure 3:**
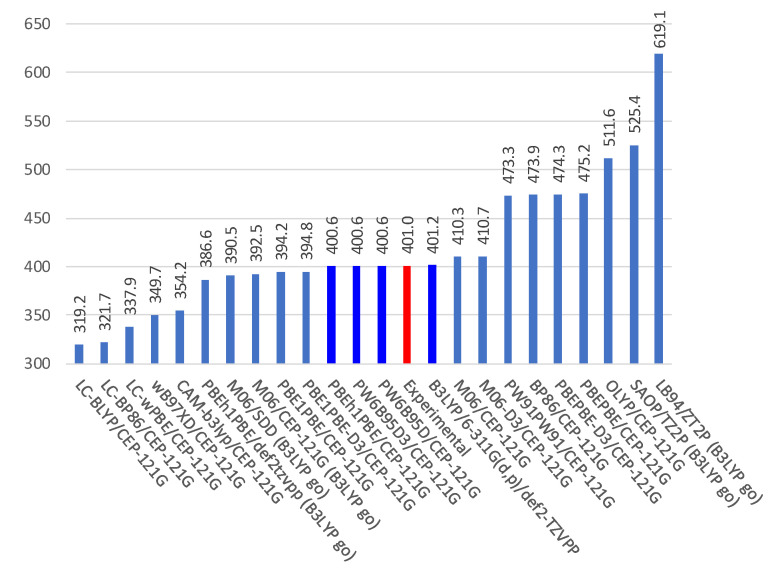
Calculated absorbance maxima (nm on *y*-axis) in the region 320–620 nm using the indicated DFT method (*x*-axis), compared to the experimental absorbance maxima 401.0 nm of Cu(HBP)_2_ (**5**), indicated in red. DFT methods in best agreement with experiment are shown in dark blue. The DFT methods used are indicated as functional/basis1/basis2 where functional is the DFT functional used, basis2 is the basis set used for Cu, and basis1 is the basis set used for C, H and O. If no basis2 is indicated, basis1 is also used for Cu. The five methods showing “B3LYP go” in brackets used the optimized B3LYP geometry in the TD-DFT calculation with the indicated method. For all the other methods, geometry optimization as well as TD-DFT calculations were done with the indicated method.

Complexes (**1**)–(**9**) exhibit experimentally a large absorbance peak in the region 340–450 nm and a small peak in the range of 550–800 nm (see Figure 2a–c with data in Table 1). Since complexes (**1**)–(**9**) showed experimental absorption in the visible wavelength range, they could be considered as dyes in DSSC. Different DFT functionals and basis set combinations were used to identify the DFT method that best reproduced the experimental UV/vis absorbance maximum 401.0 nm of Cu(HBP)_2_ (**5**), the results are shown in Figure 3. The selected long range-corrected (LC) pure functionals, as well as some hybrid functionals overestimated the excitation energy (wavelength too low), while pure functionals (PW91, BP86, PBE) and asymptotically correct XC potentials (SAOP and LB94) overestimated the excitation energy. The B3LYP functional performed best, followed by PW6B95D3 and PBEh1PB. The B3LYP optimized ground state geometry were in good agreement with an available experimental crystal structure of (**5**) [18]. 

**Figure 4:**
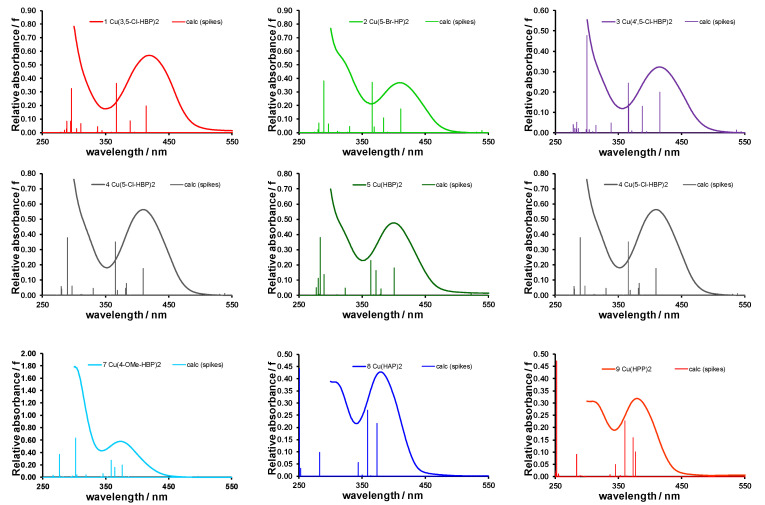
Experimental absorption spectra of the Cu(II) complexes (**1**)–(**9**) in DMSO (smooth curves) overlaid with the B3LYP/6-311G(d,p)/def2-TZVPP calculated oscillators (bar graphs of wavenumber versus the oscillator strengths).

The TDDFT simulated UV/vis spectra, obtained by selected computational methods (details of the DFT methods are provided in Section 3.1), are shown in Figure 2, with more detail of the B3LYP/6-311G(d,p)/def2-TZVPP method in Figure 4. There are 3 main bands in each of the calculated spectra, of which the indicated band 1 and band 2 correspond to the experimentally observed absorbance peaks in the regions 340–450 nm (band 2) and 550–800 nm (band 1). In the 340–450 nm (band 2) region, the calculated absorbance peaks generally consist of 2 closely overlapping peaks. The wavelength of the lowest energy oscillator in this region was used to be compared with the experimental wavelengths (Figure 3 and Table 1). The large experimental peaks of complexes (**1**)–(**9**) in the region 340–450 nm (band 2), are best reproduced by band 2 of the B3LYP/6-311G(d,p)/def2-TZVPP method with an average deviation (AD) from experiment of 2.4 nm. The PW6B95D3/CEP-121G and PBEh1PB/CEP-121G methods gave AD of band 2 values of complexes (**1**)–(**9**), from experiment values, of 6.3 and 9.1 nm, respectively. The M06/CEP-121G and M06/SDD TD-DFT methods, using the B3LYP/6-311G(d,p)/def2-TZVPP optimized geometry gave AD of band 2 values from experiment values of 9.2 and 9.8 nm, respectively. The PW6B95D3/CEP-121G and PBEh1PB/CEP-121G methods did not show any absorbance peak above 450 nm (band 1), while the B3LYP/6-311G(d,p)/def2-TZVPP method, as well as the M06/CEP-121G and M06/SDD TD-DFT methods, using the B3LYP/6-311G(d,p)/def2-TZVPP optimized geometries, did show the small experimentally observed band 1 peaks, though at a wavelength 150–200 nm lower than experimentally observed (see Figure 2). The B3LYP/6-311G(d,p)/def2-TZVPP results, the best agreement with the experiment, will thus be discussed in more detail. The B3LYP/6-311G(d,p)/def2-TZVPP calculated maximum absorption wavelengths (λ_A,max_), oscillator strength (f), and energy are summarized in Table 1 for the main absorbance peaks calculated for (**1**)–(**9**).

The B3LYP/6-311G(d,p)/def2-TZVPP method shows three obvious calculated absorption bands for complexes (**1**)–(**9**), which are located at the 240–340, 340–450 and 450–600 nm regions (see Figure 2b and Table 1). The small peaks of the UV/vis spectra in the range of 450–600 nm (band 1, the maximum wavelength peak, λ_max_), originate from LMCT transitions; see the assignments of the orbitals involved in the main transitions during the excitation of (**1**)–(**9**) in Table 2. These peaks correspond to the experimentally very small peaks observed at 550–800 nm for complexes (**1**)–(**9**) (Figure 2(aiii)). 

**Figure 5:**
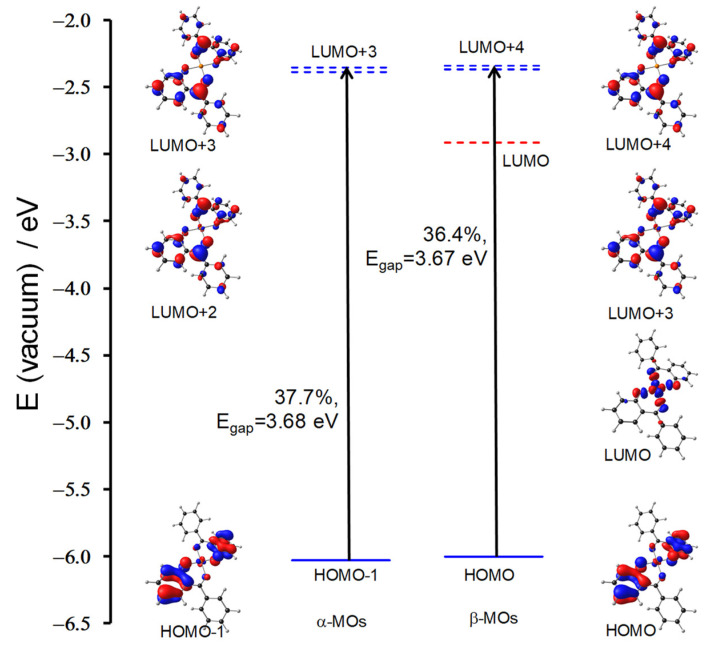
B3LYP/6-311G(d,p)/def2-TZVPP calculated Kohn−Sham MO energy level diagram illustrating the main MO transitions involved in the calculated peak at 401.2 nm, corresponding to the experimental peak at 401 nm, of (5): (i) HOMO-1 to LUMO + 3 and (ii) HOMO to LUMO + 4. The energy of occupied and unoccupied MO energy levels is shown with solid and dotted lines, respectively. Mainly copper-d-based and ligand-based MOs are shown in red and blue, respectively. Selected MOs are visualized. A contour of 0.06 e/Å^3^ was used for the orbital plot. Colour code of atoms (online version): Cu (orange), C (grey), O (red), H (white).

**Figure 6:**
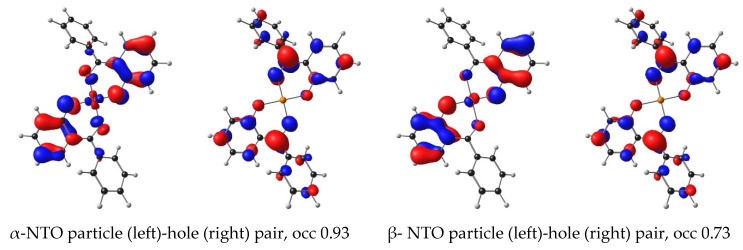
B3LYP/6-311G(d,p)/def2-TZVPP calculated NTOs involved in the calculated peak at 401.2 nm, corresponding to the experimental peak at 401 nm, of (5). The electron occupation of the occupied (particle) and unoccupied (hole) NTO pairs are indicated. A contour of 0.04 e/Å^3^ was used for the orbital plot. Colour code of atoms (online version): Cu (orange), C (grey), O (red), H (white).

The main MOs involved the transition of the maximum absorbance peak, λ_A,max_, of (**5**), are visualized is Figure 5 and summarized in Table 2 for molecules (**1**)–(**9**). The natural transition orbitals (NTOs) involved the main transitions of the λ_A,max_ peak of (**5**), are visualized in Figure 6. The NTO method provides a compact orbital representation of the orbitals involved in a transition from the electronic ground to the electronic excited state [19]. The NTO transition occurs from a small set of particle (occupied)–hole (unoccupied) pairs [20]. Both the MOs evaluation and the NTOs show than the large experimental peaks observed in the region 340–450 nm (Figure 2(aii)) that correspond to the calculated band 2 peaks (Figure 2, Table 1) in the same 340–450 nm range, are LLCT bands.

Good agreement was obtained between the TDDFT calculated oscillators of band 2 and experimental absorbance maxima in this range (indicated in Table 1 and visualized in Figure 4). Complexes with electron withdrawing ligands (complexes (**1**)–(**4**)) absorb light at higher wavelengths, while electron-donating ligands (complexes (**6**)–(**9**)) shift absorbance to lower wavelengths than (**5**) containing the unsubstituted HBP ligand. For molecules (**1**)–(**4**), the main contributions to the band 2 maximum absorbance, λ_A,max_, transition are from HOMO (β-HOMO) to LUMO + 4 (β-LUMO + 2) and from HOMO-1 (α-HOMO) to LUMO + 3 (α-LUMO + 1), with similar ligand character than observed for (**5**). However, for the complexes containing electron donating ligands molecules (**6**)–(**9**), the band 2 transitions include some LMCT character as well. The red shift to a longer wavelength of the band 2 transitions, are allocated to the involvement of the metal based LUMO in the excitation. Excitation to the LUMO contributes ca. 11% for molecules (**6**) and (**7**) and 36 or 45% for molecules (**8**) and (**9**), respectively. Molecules (**1**)–(**6**) contain 2-hydroxybenzophenone ligands, while molecule (**8**) contains a 2′-hydroxyacetophenone (HAP) and molecule (**9**) a 2′-hydroxypropiophenone (HPP) ligand.

### 2.2. DSSC Application

**Figure 7:**
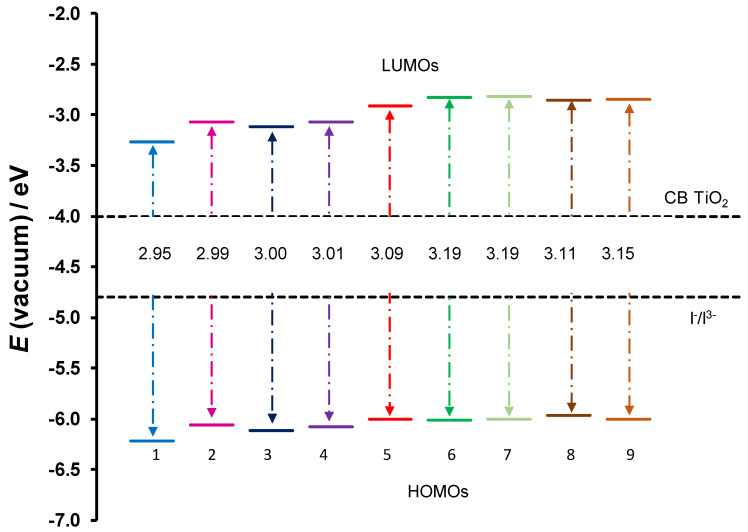
Energy levels and HOMO–LUMO gaps of the Cu(II) complexes calculated at the B3LYP/6-311G(d,p)/def2-TZVPP level in DMSO solution, compared to the CB of TiO_2_ (−4.0 eV) and the redox potential of the I^−^/I_3_^−^ electrolyte (−4.8 eV).

**Figure 8:**
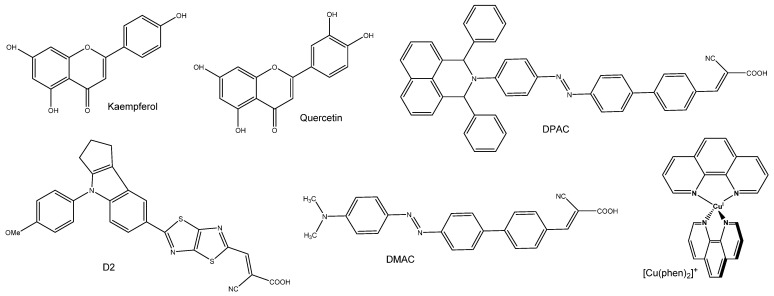
Structures of dyes theoretically investigated as photochromic dyes for DSSC.

The absorption ranges of the dyes (**1**)–(**9**) fall in the visible region, thus ensuring effective solar energy usage (see Figure 2). We therefore present and discuss theoretical calculated properties of (**1**)–(**9**), as needed for dyes in DSSC containing the (I_3_^−^/I^−^) redox mediator and TiO_2_ semiconductor. The B3LYP/6-311G(d,p)/def2-TZVPP calculated light harvesting efficiency (LHE), excited state lifetime (τ in ns), HOMO energies (E_HOMO_), LUMO energies (E_LUMO_), ΔG_inject_ (eV) and ΔG_regenerate_ (eV) values are tabulated in Table 3, with the HOMO and LUMO energies visualized in Figure 7. Table 4 gives the same calculated properties for dyes theoretically investigated as photochromic dyes for DSSC in literature [21,22,23,24] (see Figure 8 for the structures of the dyes). 

HOMO and LUMO energies are of interest since they generally are involved in the lowest energy electronic transitions. The photo-induced electron transfer from the dye to the excited state of dye is followed by electron injection into the semiconductor surface. By comparing the computed E_HOMO_ and E_LUMO_ of the dyes with the potential of the CB of the TiO_2_ semi-conductor, and with the potential of the most widely used electrolyte redox couple I^−^ /I_3_^−^ [25], respectively, the propelling force of electron injection (ΔG_inject_) and dye regeneration (ΔG_regenerate_) in DSSCs is evaluated [9]. More negative HOMO energies than the I^−^ /I_3_^−^ redox couple imply a fast regeneration of the oxidized dyes. More positive LUMO energies than the E_CB_ of TiO_2_ could ensure an effective injection of excited electrons [26]. The HOMO and LUMO energies of (**1**)–(**9**) are compared to the energy of the CB of TiO_2_ and the redox potential of the (I_3_^−^/I^−^) redox couple in Figure 7.

The B3LYP/6-311G(d,p)/def2-TZVPP calculated HOMO–LUMO gaps of complexes (**1**)–(**9**) fluctuate in the range of 2.95–3.19 eV, as shown in Figure 7. The changes in the LUMO levels (within 0.46 eV) rather than the HOMO levels (within 0.25 eV) mainly cause the differences in energy gap. Specifically, complexes (**6**) and (**7**) have the maximal HOMO–LUMO gap of 3.19 eV, while complex (**1**) has the minimum value of 2.95 eV. Comparing the unsubstituted hydroxybenzophenone Cu complex (**5**) (gap = 3.09 eV), the largest difference in the gap of 0.10 eV is observed with (**6**) and (**7**), indicating that the addition of an electron donating ester substituent, increases the LUMO level and thus leads to a larger HOMO–LUMO gap for (**6**) and (**7**). The HOMO–LUMO gap decreases significantly (up to 0.14 eV for (**1**)) with addition of electron withdrawing halide substituent groups. With the solar energy maximum occurring near 500 nm, a lower energy gap is likely to result in high photocurrents in the DSSC. This suggests that lower energy gaps are more suitable as photosensitizers in the DSSCs [27,28]. The HOMO and LUMO levels of the Cu(II) complexes fit well with the CB of TiO_2_ and the redox potential of the (I_3_^−^/I^−^) electrolyte. Figure 7 shows the HOMOs are suitably lower than the redox potential of I^−^/I_3_^−^ electrolyte (−4.8 eV) [5], which would be useful for the regeneration of the excited dyes; while the LUMOs are aptly higher than CB of TiO_2_ (4.0 eV) [29], enabling the efficient electron injection into the semiconductor.

The visible and near UV regions are the most essential region for photon-to-current conversion to obtain information about the electronic transitions and its corresponding molecular orbital properties. The LHE of the dye sensitizer should be as high as possible to maximize the photocurrent response [21,28]. The oscillator strength of the dye associated with the wavelength corresponds to the peak absorbance through intramolecular charge transfer (ICT). A high f generally results in a higher LHE, while a small HOMO–LUMO energy gap increases the light-capturing capacity and improves the efficiency of the DSSCs. The f value of the dye with alkoxy groups ((**7**), Table 1) was higher than that reported for some other dyes (Table 4), which suggested that the LHE of complex (**7**) would also be greater compared to other dyes. The LHE should be as high as possible to maximize the short-circuit current density (J_SC_) [22].

The Gibbs energy change for electron injection ∆G_inject_ affects the electron injection rate and therefore the current density J_SC_ in DSSCs. The ∆G_inject_ values of all the dyes were favourable for injection into the TiO_2_ (conduction band) CB edge. On the other hand, the redox levels of the electrolyte are higher than the ground state of the dye, leading to favourable ∆G_reg_ for regeneration of dyes. The larger the absolute values of ∆G_inject_ and ∆G_reg_, the greater will be the ease with which the charge transfers between the conduction band of the dye and the electrolyte [21].

These results indicate that the copper-based complexes (**1**)–(**9**) might be effective sensitizers for a next-generation dye-sensitized solar cell. However, since Kohn−Sham Mos involved in the main excitations are all symmetrical round copper (Figure 5), the distance between the centroid of the hole and the electron after excitation will be small. Excitation would thus mainly lead to intramolecular electron recombination with low efficiency electron injection into the semiconductor [17]. Structural modifications (e.g., mixed ligand complexes) are needed before these complexes could qualify as dyes in the framework of DSSCs.

### 2.3. Relationships Involving Experimental UV-Vis Peaks

**Figure 9:**
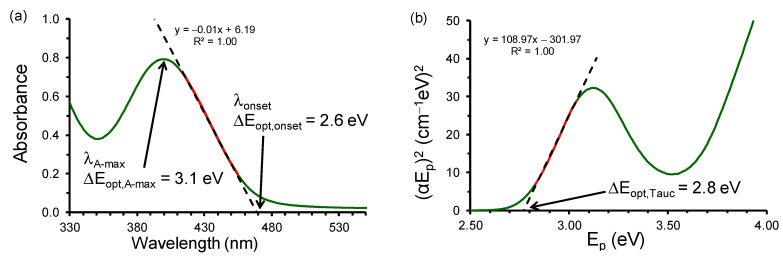
(**a**) The experimental UV of (**5**) in DMSO illustrating the determination of the onset (469 nm) and maximum absorbance (401 nm) positions that were used to calculate the experimental optical band gap (ΔE_opt_). (**b**) Tauc plot of (**5**) showing the determination of the experimental optical band gap (ΔE_opt,Tauc_).

**Figure 10:**
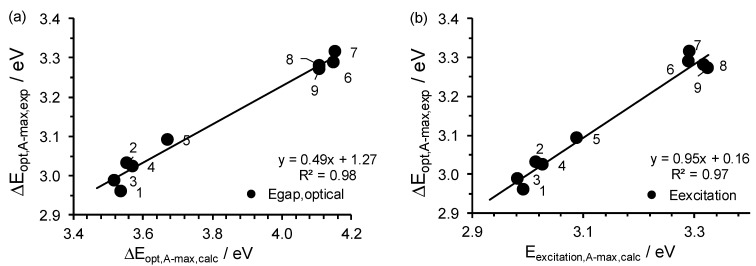
Relationship between experimentally determined optical band gap from the maximum absorbance (ΔE_opt,A-max,exp_) and (**a**) the B3LYP/6-311G(d,p)/def2-TZVPP calculated band gap between the main MOs involved in the electronic transition (ΔE_opt,A-max,calc_, average value of E_gap_ as shown in Figure 4 for (**5**)), (**b**) the excitation energy of the λ_A,max_ peak (Data in Table 5).

From the experimental optic UV-vis data, the experimental optical energy gap (ΔE_exp, optical_) related to an experimental UV-vis peak can be calculated, using different approaches such as using λ_A,max_, λ_onset_ or energy obtained from a Tauc plot, as illustrated in Figure 9a,b for molecule (**5**). The high intensity experimental peak (peak identified as band 2 in Figure 2a at 401 nm for (**5**)) was used to obtain ΔE_exp, optical, A-max_ = 3.1 eV, ΔE_exp, optical, onset_ = 2.6 eV and ΔE_exp, optical, Tauc_ = 2.8 eV for (**5**). The results are different and depend on the selected approach. Similarly, the theoretically calculated energy gap depends on the DFT method used. When calculating the theoretical energy gap, the specific Mos involved in the theoretical transition (e.g., at 401.2 nm for (**5**)), which agree with the experimental UV-Vis peak (401 nm for (**5**)), need to be used [30]; namely 37.7% HOMO-1 to LUMO + 3 (E_gap_ = 3.68 eV) and 36.4% HOMO to LUMO + 4 (E_gap_ = 3.67 eV) transitions for (**5**) (see Figure 4). The average of the E_gap_ of the main transitions involved in theoretically calculated peak (ΔE_calc, A-max_), relates to the experimentally calculated E_gap_ of the same peak (ΔE_exp, A-max_) for molecules (**1**)–(**7**), as well as to the excitation energy of the λ_A,max_ peak (see Figure 10, data in Table 5). The obtained experimentally determined E_gap_ and the DFT calculated E_gap_ do not coincide with each other, but they show similar tendencies. 

## 3. Materials and Methods

The bis(2-hydroxyphenone)copper(II) complexes (**1**)–(**9**) were synthesized and characterized as described in our previous publication [18]. UV/vis spectra were recorded on a Varian Cary 50 Conc ultra-violet/visible spectrophotometer.

### 3.1. DFT Calculations

The molecules (**1**)–(**9**) were optimized using the density functional theory (DFT) program Gaussian 16 [32] and different DFT functionals and basis set combinations. The DFT methods used are indicated as functional/basis1/basis2 where functional is the DFT functional used, basis2 is the basis set used for Cu, and basis1 is the basis set used for C, H and O. If no basis2 is indicated, basis1 is also used for Cu. The five methods showing “B3LYP go” in brackets in Figure 3 used the optimized B3LYP geometry in the TD-DFT calculation with the indicated method. For all the other methods, geometry optimization as well as TD-DFT calculations were done with the indicated method. For example, the B3LYP/6-311G(d,p)/def2-TZVPP method uses the hybrid B3LYP functional [33,34], the triple-ζ 6-311G(d,p) basis set for the lighter atoms (C, H, Cl, F, O) and the def2-TZVPP basis set [35] for the core and valence electrons of Cu. The PW6B95D3/CEP-121G and PBEh1PB/CEP-121G methods use PW6B95D3 [36] and PBEh1PBE [37] functionals, respectively, both with the CEP-121G basis set [38,39,40] for all atoms. TD-DFT calculations were performed on molecules (**1**)–(**9**) [41,42,43], using the same level of theory as for the optimization, except when indicated that the B3LYP optimized geometries were used. 

All calculations were performed in DMSO as implicit solvent, the experimental solvent used for the UV-vis spectra. The improved IEF-PCM solvation model, the integral equation formalism (IEF) version of the Polarizable Continuum Model (PCM), was used to model solvation effects [44,45]. The input coordinates for the compounds were constructed using Chemcraft [46]. 

From the TD-DFT results, the following properties related to DSSC were obtained or calculated:

1. λ_max_ with the corresponding calculated transition energy *E* (cm^−1^) and the calculated oscillator strength (*f*) of the excited state;
2. Excited state lifetime (τ in ns) [16];
3. Light harvesting efficiency (LHE) [47,48];
4. The injection driving force of a dye (Δ*G*_inject_) [9];
5. The regeneration driving force of a dye (Δ*G*_regenerate_) [9];
6. Using the equation below:

τ (in s) = 1.499/(*f E*^2^) (1)

(2) $$\mathrm{LHE}=1-10^{-f}$$

(3) $$\Delta G_{\mathrm{inject}}=E_{\mathrm{dye}}^{*}-E_{\mathrm{CB}}=E_{\mathrm{dye}}-E_{\lambda_{\max}}-E_{\mathrm{CB}}\;$$

(4) $$\Delta G_{\mathrm{regenerate}}=\left(E_{electrolyte}\;\right)-\left(E_{\mathrm{dye}}\right)\;$$

where $E_{\mathrm{dye}}^{*}$ is the oxidation potential of the excited dye (approximated by energy difference $E_{\mathrm{dye}}-E_{\lambda_{\max}}$), *E*_dye_ is the oxidation potential of the dye (approximated by HOMO energy), *E*_CB_ is the reduction potential of the conduction band edge (TiO_2_ with *E*_CB_ = −4.0 eV vs. vacuum or −0.5 eV vs. NHE [29] is used in this study), $E_{\lambda_{\max}}\;$is the electronic vertical transition energy corresponding to λ_max_ potential, and $E_{electrolyte}\;$is the redox potential of the electrolyte (the I^−^/I_3_^−^ redox couple is used in this study, with $E_{I^{-}/I_{3}^{-}}\;$= −4.8 eV vs. vacuum or 0.3 eV vs. NHE) [25].

### 3.2. Calculations Involving Experimental Data

From the experimental UV-vis, the experimental band gap energy can be calculated from the formula ΔE_opt_ = hc/λ = 1240/λ, where the wavelength λ is obtained from (i) the maximum absorbance wavelength λ_max_, (ii) the λ_onset_ or (iii) from a Tauc plot denoting the onset of absorption [49] (see Section 2.3). The Tauc plot of (αE_p_)^2^ versus E_p_ is according to the following equation [50,51,52,53]:

αE_p_ = K(E_p_ − E_g_′)^1/2^ (5)

where *α* represents the absorption coefficient, K is a constant, E_p_ is photon energy and E_g_′ is the band-gap energy. Values for *α* and E_p_ are determined by:

*α* = 2.303 A/d (6)

E_p_ = hc/λ (7)

where A is the absorption (arbitrary units) and d is the sample thickness, which for a cuvette is 1 cm, h Planck’s constant with hc = 1240 eV and λ representing the wavelength in nm. The band-gap energy, E_g_′_,_ was determined by linear extrapolation of absorption edges on the graph of (αE_p_)^2^ versus E_p_ to the *x*-axis where y = 0. 

## 4. Conclusions

The lowest energy excitation of the Cu(HBP)_2_ complexes, involving a ligand-based MO and a metal based LUMO, are of LMCT character, while all other low energy excitations that do not involve the LUMO, will be LLCT bands. TDDFT results and properties related to DSSC (λ_A,max_, τ, LHE, Δ*G*_inject_ and Δ*G*_regenerate_) indicated that these Cu(HBP)_2_ complexes show promising performance, and with structural modifications can be made suitable for applications in the next-generation DSSCs. 

## Figures and Tables

**Table 1:** B3LYP/6-311G(d,p)/def2-TZVPP calculated absorbance maximum wavelength (λ_max,calc_), oscillator strengths (f), as well as the experimental maximum wavelength (λ_max_) and maximum absorbance (λ_A,max_) peaks.

		Calculated								Experimental	
	Complex	(λ_A,max,calc_) (nm) with f (Calculated Oscillator Strength) in Brackets								λ_A,max_ (nm)	λ_max_ (nm)
		Band 3		Band 2a		Band 2b		Band 1		exp	exp
**1**	Cu(3,5-Cl)_2_	296.1	(0.33)	366.7	(0.36);	414.2	(0.20)	552.9	(0.02)	419	750
**2**	Cu(5-Br)_2_	289.6	(0.38)	366.1	(0.37);	411.1	(0.17)	539.6	(0.01)	409	720
**3**	Cu(4’,5-Cl)_2_	300.0	(0.48)	365.5	(0.24);	415.7	(0.20)	536.6	(0.01)	415	723
**4**	Cu(5-Cl)_2_	289.7	(0.38)	365.7	(0.35);	409.4	(0.18)	538.6	(0.01)	410	707
**5**	Cu(HBP)_2_	284.2	(0.38)	364.0	(0.23);	401.2	(0.18)	522.4	(0.01)	401	674
**6**	Cu(4-allyloxy)_2_	302.1	(0.51)	359.1	(0.27);	376.8	(0.19)	498.0	(0.01)	377	660
**7**	Cu(4-OMe)_2_	302.2	(0.63)	358.8	(0.27);	376.6	(0.19)	497.1	(0.01)	374	658
**8**	Cu(HAP)_2_	250.7	(0.44)	358.8	(0.27);	373.6	(0.22)	489.6	(0.00)	378	655
**9**	Cu(HPP)_2_	252.0	(0.47)	360.1	(0.23);	372.9	(0.16)			379	650

**Table 2:** Computed maximum wavelengths (λ_max,calc_), transition energy (ΔE in eV), oscillator strength (f) and assignments of main transitions involved in the excitation of Cu(L)_2_ complexes (**1**)–(**9**).

No.		E (eV)	λ_max,calc_ (nm)	f	from	to	% Contribution	Assignment	ΔE_calc_ (eV) Transition
1	Cu(3,5-Cl)_2_	2.99	414.15	0.20	HOMO−1	LUMO + 3	42.5	LLCT	3.54
					HOMO	LUMO + 4	41.7	LLCT	3.54
2	Cu(5-Br)_2_	3.02	411.08	0.17	HOMO−1	LUMO + 3	41.3	LLCT	3.56
					HOMO	LUMO + 4	41.3	LLCT	3.55
3	Cu(4’,5-Cl)_2_	2.98	415.68	0.20	HOMO−1	LUMO + 3	41.8	LLCT	3.52
					HOMO	LUMO + 4	42.1	LLCT	3.51
4	Cu(5-Cl)_2_	3.03	409.39	0.18	HOMO−1	LUMO + 3	41.1	LLCT	3.58
					HOMO	LUMO + 4	41.2	LLCT	3.57
5	Cu(HBP)_2_	2.37	522.39	0.97	HOMO−2	LUMO	87.59	LMCT	3.31
5	Cu(HBP)_2_	3.09	401.21	0.18	HOMO−1	LUMO + 3	37.7	LLCT	3.68
					HOMO	LUMO + 4	36.4	LLCT	3.67
6	Cu(4-allyloxy)_2_	3.29	376.80	0.19	HOMO−1	LUMO + 3	25.6	LLCT	3.90
					HOMO−17	LUMO	10.9	LMCT	4.66
					HOMO	LUMO + 4	28.8	LLCT	3.89
7	Cu(4-OMe)_2_	3.29	376.61	0.19	HOMO−1	LUMO + 3	26.9	LLCT	3.90
					HOMO−17	LUMO	11.9	LMCT	4.67
					HOMO	LUMO + 4	29.0	LLCT	3.89
8	Cu(HAP)_2_	3.32	373.63	0.22	HOMO−1	LUMO + 3	21.3	LLCT	3.93
					HOMO−5	LUMO	44.6	LMCT	4.50
					HOMO	LUMO + 4	26.2	LLCT	3.89
9	Cu(HPP)_2_	3.33	372.86	0.16	HOMO−1	LUMO + 3	17.7	LLCT	3.95
					HOMO−5	LUMO	35.9	LMCT	4.47
					HOMO	LUMO + 5	21.0	LLCT	3.91

**Table 3:** B3LYP/6-311G(d,p)/def2-TZVPP calculated light harvesting efficiency (LHE), excited state lifetime (τ), E_HOMO_, E_LUMO_, ΔG_inject_ and ΔG_regenerate_.

	Complex	LHE				τ (ns)				E_HOMO_	E_LUMO_	∆G_inject_	∆G_reg_
		Band 3	Band 2a	Band 2b	Band 1	Band 3	Band 2a	Band 2b	Band 1	(eV)	(eV)	(eV) (Band 2b)	(eV)
**1**	Cu(3,5-Cl)_2_	0.53	0.57	0.36	0.05	4.0	5.6	13.1	206.4	−3.27	−6.22	0.78	1.42
**2**	Cu(5-Br)_2_	0.58	0.57	0.33	0.03	3.3	5.4	14.6	294.9	−3.07	−6.06	0.96	1.26
**3**	Cu(4’,5-Cl)_2_	0.67	0.43	0.37	0.03	2.8	8.2	13.0	317.4	−3.11	−6.11	0.87	1.31
**4**	Cu(5-Cl)_2_	0.58	0.55	0.34	0.03	3.3	5.7	14.2	381.4	−3.07	−6.08	0.95	1.28
**5**	Cu(HBP)_2_	0.58	0.41	0.34	0.03	3.2	8.6	13.4	292.2	−3.07	−6.08	1.01	1.28
**6**	Cu(4-allyloxy)_2_	0.69	0.47	0.35	0.03	2.7	7.1	11.5	295.1	−2.83	−6.01	1.28	1.21
**7**	Cu(4-OMe)_2_	0.76	0.46	0.36	0.03	2.2	7.1	10.9	319.3	−2.82	−6.01	1.28	1.21
**8**	Cu(HAP)_2_	0.64	0.46	0.39	0.00	2.1	7.1	9.7	35,931.1	−2.86	−5.97	1.35	1.17
**9**	Cu(HPP)_2_	0.66	0.41	0.31	0.00	2.0	8.6	13.1		−2.85	−6.00	1.32	1.20

**Table 4:** Published values of absorbance maximum wavelength (λ_max,calc_), oscillator strengths (f), light harvesting efficiency (LHE), excited state lifetime (τ), E_HOMO_, E_LUMO_, ΔG_inject_ and ΔG_regenerate_ for the indicated dyes theoretically investigated as photochromic dyes for DSSC (structures in Figure 8).

Dye	λ_max,calc_ (nm)	f	LHE	τ (ns)	E_HOMO_ (eV)	E_LUMO_ (eV)	HOMO–LUMO Gap (eV)	∆G_inject_ (eV)	∆G_reg_ (eV)	Ref
Quercetin	352	0.54	0.71	2.17	−5.69	−1.71	3.97	1.83	0.84	[21]
Quercetin	356	0.40	0.60	2.35	5.83			1.65		[23]
Kaempferol	365	0.59	0.75	2.00	−5.39	−1.58	3.81	2.20	0.54	[21]
DMAC (trans)	430	2.05	0.99	1.43	−5.23	−2.76	2.48	1.65	0.43	[22]
DMAC (cis)	347	0.99	0.90	1.99	−5.23	−2.69	2.54	1.40	0.43	[22]
DPAC (trans)	440	1.80	0.95	1.52	−5.23	−2.80	2.42	1.66	0.43	[22]
DPAC (cis)	341	0.80	0.84	2.57	−5.20	−2.72	2.48	1.35	0.40	[22]
D2	433	1.48	0.97		5.57	2.71		1.29		[24]
[Cu(phen)_2_]^+^	443	0.250	0.44	11.8			3.88	0.78	1.22	[17]

**Table 5:** B3LYP/6-311G(d,p)/def2-TZVPP E_gap_ and experimentally determined E_gap_ (from UV-vis absorbance maximum wavelength λ_A,max_).

No.	Complex	λ_A,max_	E_excitation_	ΔE_DFT-calc, A-max_^a^	ΔE_exp, optical, A-max_
1	Cu(3,5-Cl)_2_	414.2	2.99	3.54	2.96
2	Cu(5-Br)_2_	411.1	3.02	3.55	3.03
3	Cu(4’,5-Cl)_2_	415.7	2.98	3.52	2.99
4	Cu(5-Cl)_2_	409.4	3.03	3.57	3.02
5	Cu(HBP)_2_	401.2	3.09	3.67	3.09
6	Cu(4-allyloxy)_2_	376.8	3.29	3.89	3.29
7	Cu(4-OMe)_2_	376.6	3.29	3.90	3.32
8	Cu(HAP)_2_	373.6	3.32	4.11	3.28
9	Cu(HPP)_2_	372.9	3.33	4.11	3.27

^a^ Average value of main transitions involved in the excitation. E_gap_ value obtained from Kohn−Sham MO energies, differs from the E_excitation_, since Kohn−Sham MO energies gaps are approximations to excitation energies [31].

## Data Availability

The data presented in this study are available on request from the corresponding author.

## Sample Availability

Samples of the compounds are not available from the authors.
